# High nutrition literacy linked with low frequency of take-out food consumption in chinese college students

**DOI:** 10.1186/s12889-023-16078-9

**Published:** 2023-06-13

**Authors:** Qi Qi, Qing Sun, Ling Yang, Yan Cui, Jing Du, Huaqing Liu

**Affiliations:** School of Public Health, Bengbu Medical College, Bengbu, Anhui 233030 China

**Keywords:** Nutrition literacy, Healthy literacy, Take-out food, College students, China

## Abstract

**Background:**

The excessive consumption of take-out food has increased the risk of nutrition-related chronic diseases. Nutrition literacy (NL) is an important influencing factor of food choice. This study aimed to explore the relationship between nutrition literacy and take-out food consumption.

**Methods:**

A cross-sectional study was conducted on 2130 college students in Bengbu, China. A self-reported questionnaire that included demographic information, lifestyle behavior, take-out food consumption, and nutrition literacy scale was used. Ordinal logistic regression models were used to analyze the association between nutrition literacy and take-out food consumption.

**Results:**

Of the students surveyed, 61.5% consumed take-out food at least once a week. NL was significantly associated with the frequency of take-out food consumption ≥ 4 times/week (OR = 0.995, 95% CI = 0.990-1.000); the difference specifically was discovered for applying skills, interactive skills, and critical skills. Moreover, students with high level NL ate less (Spicy) hot pot (OR = 0.996, 95% CI = 0.992-1.000), but more vegetable and fruit salad (OR = 1.009, 95% CI = 1.002–1.015).

**Conclusions:**

NL, especially in applying skills, interactive skills, and critical skills, is not only associated with consumption frequency of take-out food among college students, but also links with types of take-out food consumption. Our findings emphasize that targeted interventions on nutritional skills literacy should be needed to improve dietary behaviors for student’s good health.

**Supplementary Information:**

The online version contains supplementary material available at 10.1186/s12889-023-16078-9.

## Introduction

The rapid development of the fast-food industry has changed people’s food consumption patterns in the past few decades. Take-out food consumption has become an essential component of people’s diet. Studies have displayed the increasing frequency of takeout consumption in western countries. A cross-sectional survey in the United Kingdom found that approximately 21% of the adults and children ate take-away meals at home at least once a week [1]. The Health and Nutrition Examination Survey showed that more than one-third (36.3%) of US residents consumed fast food on a given day during 2015–2018 [2]. A longitudinal study demonstrated that the proportion of Australians eating takeaway meals once or more per week increased from 35.5 to 44.1% over the five years [3]. China has the largest number of consumers who experienced online takeout food globally [4, 5].

Take-out food was defined as food items from fast-food outlets with convenient delivery service, carryout food options, and payment prior to the receipt of food [6, 7]. However, take-out food is energy-dense, nutrient-poor, and rich in salt, sugar, fat [6]. Takeout food consumption had adverse effects on public health, and most previous studies have reported that more frequent consumption of takeaway meals increased the risk of obesity [8–10], hypertension [11], type 2 diabetes, and coronary heart disease [12]. Moreover, people may ingest microplastics through take-out food containers and affect health [13]. Food safety issues related to the preparation process and sanitary conditions of take-out food have also not been fundamentally corrected [13, 14]. As a special eating behavior, take-out food consumption is a concern. It is affected by many factors (personal factors, economic factors and social environmental factors, etc.) [4].

College students who are transitioning from adolescence to young adulthood have greater needs for various nutrients and are at a critical period in which they are forming independent eating habits that can continue throughout life. Facing with academic pressure and employment pressure, college students are more likely to choose convenient take-out food [15, 16]. In a cross-sectional study of college students attending Beijing University in China, all participants surveyed consumed take-out food per week; moreover, nearly one-third of them consumed it 9–15 times per week [17].

Nutrition literacy (NL) is, regarded as a specific form of health literacy [18], defined as the capability to obtain, process, and understand nutrition information and skills to make suitable nutritional decisions [19–21], which is an important determinant of eating behavior [22]. People with high level of NL would follow the dietary guidelines to choose a healthy diet, while people with low level of NL might not have the ability to eat properly, resulting in poor diet quality [23]. College students with low nutritional knowledge, attitude, and practices scores were more likely to eat fast food [24]. High nutrition knowledge was positively associated with high consumption of fruit and vegetable [23]. Faced with the widespread pseudoscience of nutrition and diet, college students were often confused and skeptical. Moreover, a high level of critical nutrition literacy was required to distinguish scientific and reliable information from the complex nutritional information environment [25]. However, a randomized controlled study showed that traditional nutrition education only increases nutrition knowledge, but failed to change eating behavior, leading to the separation of knowledge and practice among college students [26]. Therefore, Nutrition and health education should not only be directed at improving people’s knowledge, but also understanding and capacity to act. In addition, and a recent cross-sectional study reported that there was an inverse association between macronutrient literacy and unhealthy food habits [27], whereas fast food consumption is one of the unhealthy food habits.

Existing studies mainly focused on the relationship between take-out food and disease, and the impact of environmental, demographic, and other factors on take-out consumption [4]. However, to date, the relationship of NL with takeout food consumption is still unclear. The purpose of our study is aimed to investigate the relationship between NL and takeout food consumption among college students.

## Methods

### Study design

This study is a cross-sectional study, which was conducted from April to June 2021 to explore the association between NL and take-out food consumption in Bengbu, China. All students were gathered in the classroom and a self-designed structured questionnaire was used to collect data by self-administered questionnaires survey in person on the spot. All participants were notified that participation was voluntary, and signed informed consent was obtained. This study was approved by the Ethics Committee of Bengbu Medical College.

### Participants

Participants were recruited with the method of stratified cluster sampling according to the type of universities and the levels of education. Firstly, among six universities (one medical and five non-medical) in Bengbu, two universities (medical and non-medical) were selected by convenience sampling, which have equivalent admission scores at national college entrance examination. Secondly, eight classes were randomly selected in each grade, and all students in these classes were asked to participate in the survey. A total of 2,190 students finished the survey at two universities. Finally, a total of 2,130 students were analyzed in this study, after the exclusion of 60 (2.9%) because of invalid responses with missing nutrition literacy data (n = 49) and take-out food consumption data (n = 11).

### Take-out food consumption

Take-out food consumption was identified by self-designed questionnaire, including the frequency and types of take-out food consumption. The frequency of take-out food consumption was assessed by the following two questions: “Have you ordered takeout foods in the past month?” and “How often do you order takeout foods?”. Response to first question was “yes” or “no”, and response to second question included “< 1 time /week”, “1~3 times /week”, or “≥ 4 times /week”. Types of take-out food consumption was assessed by the following question: “What kind of take-out food do you prefer?” Responses included “Chinese dishes with rice”, “Western fast food”, “(Spicy) hot pot”, “Barbecue skewers”, “Pastries/ drinks”, and “Vegetable and fruit salad”.

### Nutrition literacy assessment

The NL questionnaire of 43 items, which was developed by Delphi consultation and has been validated in the Chinese adult population and university students with a good validity [28–30], was used to assess the participants’ nutrition literacy from two distinctive domains of nutrition cognitive and nutrition skills (the NL questionnaire and scoring manual has been provided in Additional file 2). The nutrition cognitive domain included two dimensions: “knowledge” (7 items) and “understanding” (5 items). The nutrition skills domain consisted of four dimensions: “obtaining skills” (5 items), “applying skills” (11 items), “interactive skills” (9 items), and “critical skills” (6 items). Each item was scored ranging from 1 to 5 on a five-point Likert type scale (for agreement: 1 = strongly disagreeable, 2 = disagreeable, 3 = neutral, 4 = agreeable, 5 = strongly agreeable; or for match: 1 = strongly unmatched, 2 = unmatched, 3 = neutral, 4 = matched, 5 = strongly matched). Then the total scores were calculated from the 43 items, with a higher score indicating a higher NL level. The NL levels were classified into four categories according to the interquartile of the scores.

### Adjustment covariates

Given demographic characteristics and lifestyle behaviors were associated with take-out food consumption in previous studies [4, 17] and could be potential confounding factors, we adjusted these covariates. Demographic characteristics were gender (0 = male, 1 = female), major (0 = medical students, 1 = non-medical students), academic year (0 = freshman, 1 = sophomore, 2 = junior, 3 = senior), birthplace (0 = urban,1 = rural), monthly expenses (RMB) (0 = < 1000, 1 = 1000~, 2 = 1500~) and formal education in nutrition (0 = no, 1 = yes). Lifestyle behaviors were Sitting time (h/day) (0 = < 4, 1 = 4~, 2 = ≥ 6), physical activity (0 = rare, 1 = occasional, 2 = frequent) and online time (h/day) (0 = < 5, 1 = 5~, 2 = ≥ 7).

### Statistical analysis

Descriptive statistics were expressed as mean ± standard deviation for continuous variable, and were expressed as frequencies and percentages for categorical variables. Chi-square test was applied to compare differences in groups for categorical variables. Multinomial logistic regression models were used to access the associations of NL and the frequencies of take-out food consumption by calculating the odds ratios (OR) and 95% confidence intervals (CIs), and multivariate logistic regression models were used to access the associations of NL and types of take-out food consumption. All analysis was conducted using Stata16.0. *P* < 0.05 (two-sided) was considered statistically significant.

## Results

### Basic characteristics

The demographic characteristics and lifestyle behavior variables are shown in Table 1. There were 2,130 undergraduate students (mean [SD] age: 20.9 [1.6] years) enrolled in the study. Among them, 34.6% (n = 736) were male, 47.0% (n = 1002) were medical, 27.9% (n = 594) were freshmen, 30.4% (n = 647) were urban, 44.6% (n = 949) of the students rarely participated in physical activity, 25.2% (n = 536) of the students’ monthly expenses were more than 1500 RMB. In addition, more than a quarter of the students (25.1%, n = 534) reported sitting time of 4 h or less, and over one-third (34.9%, n = 743) online time of 5 h or less. The score of total nutritional literacy was 151.3 ± 24.8, with 29.3 ± 5.8 in knowledge, 18.5 ± 3.9 in understanding, 17.2 ± 3.7 in obtaining skills, 36.1 ± 7.5 in applying skills, 30.3 ± 6.2 in interactive skills, 20.0 ± 4.5 in critical skills.

**Table 1:** Frequency of take-out food consumption among different characteristics

Variables	N	< 1 time /week	1~3 times /week	≥ 4 times /week	*χ* ^*2*^	*P*
***Total***	2130(100.0)	819 (38.5)	790 (37.0)	521 (24.5)		
***Age group(years)***						
16–21	1590(74.6)	621(39.1)	594(37.4)	375(23.6)	2.685	0.261
22–27	540(25.4)	198(36.7)	196(36.3)	146(27.0)		
***Gender***						
Male	736 (34.6)	246 (33.4)	228 (31.0)	262 (35.6)	75.747	< 0.001
Female	1394 (65.4)	573 (41.1)	562 (40.3)	259 (18.6)		
***Major***						
Medical students	1002 (47.0)	387 (38.6)	328 (32.7)	287 (28.6)	23.221	< 0.001
Non-medical students	1128 (53.0)	432 (38.3)	462 (41.0)	234 (20.7)		
***Academic year***						
Freshman	594 (27.9)	183 (30.8)	244 (41.1)	167 (28.1)	54.867	< 0.001
Sophomore	474 (22.3)	225 (47.5)	170 (35.9)	79 (16.7)		
Junior	491 (23.1)	219 (44.6)	167 (34.0)	105 (21.4)		
Senior	571 (26.8)	192 (33.6)	209 (36.6)	170 (29.8)		
***Birthplace***						
Urban	647 (30.4)	229 (35.4)	237 (36.6)	181 (28.0)	7.005	0.030
Rural	1483 (69.6)	590 (39.8)	553 (37.3)	340 (22.9)		
***Monthly expenses (RMB)***						
< 1000	275 (12.9)	143 (52.0)	75 (27.3)	57 (20.7)	36.957	< 0.001
1000~	1319 (61.9)	512 (38.8)	497 (37.7)	310 (23.5)		
1500~	536 (25.2)	164 (30.6)	218 (40.7)	154 (28.7)		
***Physical activity***						
Rare	949(44.6)	371(39.1)	320(33.7)	258(27.2)	13.150	0.011
Occasional	787(36.9)	287(36.5)	324(41.2)	176(22.4)		
Frequent	394(18.5)	161(40.9)	146(37.1)	87(22.1)		
***Sitting time (h/day)***						
< 4	534 (25.1)	217 (40.6)	172 (32.2)	145 (27.2)	13.041	0.011
4~	597 (28.0)	212 (35.5)	253 (42.4)	132 (22.1)		
≥ 6	999 (46.9)	390 (39.0)	365 (36.5)	244 (24.4)		
***Online time (h/day)***						
< 5	743 (34.9)	359 (48.3)	250 (33.6)	134 (18.0)	56.720	< 0.001
5~	715 (33.6)	257 (35.9)	264 (36.9)	194 (27.1)		
≥ 7	672 (31.5)	203 (30.2)	276 (41.1))	193 (28.7)		

Overall, 61.5% of the students consumed take-out food at least once a week. As shown in Table 1, the prevalence of take-out food consumption for < 1time, 1–3 times, and ≥ 4 times per week were 38.5%, 37.0%, and 24.5%, respectively. The frequency of take-out food consumption among males, medical students, and urban residents group was significantly higher than their counterpart. Moreover, participants with higher monthly expenses, less physical activity or longer online time had higher take-out consumption, and those with the lowest sitting time (< 4 h/d) had lower take-out consumption. In addition, freshmen and seniors had higher prevalence rates on take-out food consumption, compared to sophomores and juniors.

### Frequency of take-out food consumption according to NL

Table 2 demonstrated the results of the frequency of take-out food consumption according to NL in different dimensions. The fourth quartile with the highest NL showed a significantly lowest frequency of take-out food consumption (*P <* 0.05). The third quartile and fourth quartile with higher nutrition skills domain showed a significantly lower take-out food consumption than the first quartile with the lowest nutrition skills domain (*P <* 0.05), while the nutrition cognitive domain was not associated with takeout consumption. When compared to those with lowest nutrition literacy in different dimensions of nutrition skills, those who had higher nutrition literacy (i.e., those below 25th compared with above 25th percentile) were less likely to consume take-out food (*P <* 0.05), except for obtaining skills (*P >* 0.05). In the two dimensions of nutrition cognitive domain, the first quartile with the lowest knowledge NL demonstrated a significantly higher frequency of take-out consumption than the fourth quartile with the highest knowledge NL (*P <* 0.05), while the understanding NL was not significantly associated with take-out food consumption (P > 0.05).

**Table 2:** Frequency of take-out food consumption according to NL

Variables	N	< 1time /week	1~3 times /week	≥ 4times /week	*χ* ^*2*^	*P*
*NL*						
Q1	567(26.6)	214(37.7)	202(35.6)	151(26.6)	19.365	0.004
Q2	522(24.5)	172(33.0)	207(39.7)	143(27.4)		
Q3	512(24.0)	195(38.1)	199(38.9)	118(23.0)		
Q4	529(24.8)	238(45.0)	182(34.4)	109(20.6)		
***Nutrition cognition***						
Q1	604(28.4)	227(37.6)	215(35.6)	162(26.8)	8.852	0.182
Q2	560(26.3)	214(38.2)	218(38.9)	128(22.9)		
Q3	453(21.3)	181(40.0)	179(39.5)	93(20.5)		
Q4	513(24.1)	197(38.4)	178(34.7)	138(26.9)		
***Nutrition skills***						
Q1	647(30.4)	233(36.0)	236(36.5)	178(27.5)	16.922	0.010
Q2	424(19.9)	141(33.3)	170(40.1)	113(26.7)		
Q3	537(25.2)	217(40.4)	196(36.5)	124(23.1)		
Q4	522(24.5)	228(43.7)	188(36.0)	106(20.3)		
***Knowledge***						
Q1	1016(47.7)	384(37.8)	372(36.6)	260(25.6)	20.413	0.002
Q2	72(3.4)	29(40.3)	29(40.3)	14(19.4)		
Q3	524(24.6)	191(36.5)	228(43.5)	105(20.0)		
Q4	518(24.3)	215(41.5)	161(31.1)	142(27.4)		
***Understanding***						
Q1	561(26.3)	226(40.3)	196(34.9)	139(24.8)	7.063	0.315
Q2	616(28.9)	242(39.3)	243(39.4)	131(21.3)		
Q3	510(23.9)	191(37.5)	185(36.3)	134(26.3)		
Q4	443(20.8)	160(36.1)	166(37.5)	117(26.4)		
***Obtaining skills***						
Q1	754(35.4)	293(38.9)	273(36.2)	188(24.9)	0.568	0.997
Q2	451(21.2)	172(38.1)	172(38.1)	107(23.7)		
Q3	643(30.2)	246(38.3)	241(37.5)	156(24.3)		
Q4	282(13.2)	108(38.3)	104(36.9)	70(24.8)		
***Applying skills***						
Q1	595(27.9)	191(32.1)	221(37.1)	183(30.8)	38.953	< 0.001
Q2	504(23.7)	185(36.7)	197(39.1)	122(24.2)		
Q3	523(24.6)	200(38.2)	197(37.7)	126(24.1)		
Q4	508(23.8)	243(47.8)	175(34.4)	90(17.7)		
***Interactive skills***						
Q1	794(37.3)	262(33.0)	304(38.3)	228(28.7)	21.324	0.002
Q2	277(13.0)	109(39.4)	102(36.8)	66(23.8)		
Q3	561(26.3)	231(41.2)	207(36.9)	123(21.9)		
Q4	498(23.4)	217(43.6)	177(35.5)	104(20.9)		
***Critical skills***						
Q1	955(44.8)	332(34.8)	371(38.8)	252(26.4)	15.289	0.018
Q2	191(9.0)	82(42.9)	67(35.1)	42(22.0)		
Q3	483(22.7)	182(37.7)	182(37.7)	119(24.6)		
Q4	501(23.5)	223(44.5)	170(33.9)	108(21.6)		

### Association of NL with the frequency of take-out food consumption

Overall, adjusting for age group, gender, major, academic year, birthplace, monthly expenses, physical activity, sitting time, and online time, NL was significantly associated with the frequency of take-out food consumption ≥ 4 times/week (OR = 0.995, 95% CI = 0.990-1.000). In addition, this relationship was observed in nutrition skills domain (OR = 0.990, 95% CI = 0.984–0.996) including applying skills (OR = 0.962, 95% CI = 0.946–0.978), interactive skills (OR = 0.975, 95% CI = 0.956–0.994) and critical skills (OR = 0.968, 95% CI = 0.943–0.994), but not for obtaining skills (OR = 1.005, 95% CI = 0.974–1.037). Take-out food consumption was not associated with the nutrition cognitive domain containing knowledge and understanding (Table 3).

**Table 3:** Association between nutritional literacy and the frequency of take-out food consumption

Variables	1~3 times/week		≥ 4times/week	
	*OR(95% CI)*	*P*	*OR(95% CI)*	*P*
NL	0.998(0.994–1.003)	0.480	0.995(0.990-1.000)	0.043
Nutrition cognitive	1.007(0.995–1.019)	0.280	1.009(0.995–1.023)	0.214
Nutrition skills	0.996(0.991–1.002)	0.161	0.990(0.984–0.996)	0.002
Knowledge	1.005(0.987–1.023)	0.590	1.005(0.985–1.026)	0.593
Understanding	1.022(0.995–1.050)	0.118	1.031(0.999–1.063)	0.054
Obtaining skills	1.012(0.985–1.041)	0.383	1.005 (0.974–1.037)	0.751
Applying skills	0.979(0.965–0.993)	0.004	0.962(0.946–0.978)	< 0.001
Interactive skills	0.994(0.977–1.011)	0.465	0.975(0.956–0.994)	0.009
Critical skills	0.989(0.967–1.013)	0.365	0.968(0.943–0.994)	0.016

The OR (95% CI) was calculated by the multinomial logistic regression adjusting for age group, gender, major, academic year, birthplace, monthly expenses, physical activity, sitting time and online time. The frequency of take-out food consumption < 1time /week is referred as the reference group

### Association of total NL with types of take-out food consumption

Types of take-out food consumption were displayed in Additional file 1. Chinese dishes with rice were the most common type of take-out food consumption (53.1%), followed by (spicy) hot pot (33.1%), pastries/ drinks (25.1%), and western fast food (22.7%). In addition, only 9.9% of the students consumed vegetable and fruit salad.

We further investigated the association between NL and types of take-out food consumption, as shown in Table 4. After adjusting for potential confounders, a mixed association was observed between NL and types of take-out food consumption. High level NL ate less (Spicy) hot pot (OR = 0.996, 95% CI = 0.992-1.000), but more vegetable and fruit salad (OR = 1.009, 95% CI = 1.002–1.015). However, no significant association was observed between NL and Chinese dishes with rice, western fast food, barbecue skewers and pastries/drinks.

**Table 4:** Association between NL and types of take-out food consumption by multivariate logistic regression

Variables	*OR(95% CI)*	*P*
Chinese dishes with rice	1.000(0.996–1.004)	0.946
Western fast food	1.000(0.996–1.005)	0.874
(Spicy) hot pot	0.996(0.992-1.000)	0.031
Barbecue skewers	0.997(0.992–1.002)	0.314
Pastries/ drinks	0.999(0.995–1.004)	0.708
Vegetable and fruit salad	1.009(1.002–1.015)	0.007

The OR (95% CI) was calculated by the multivariate logistic regression adjusting for age group, gender, major, academic year, birthplace, monthly expenses, physical activity, sitting time and online time

## Discussion

This study is the first time to examine the association between NL and takeout food consumption among students in China. The findings showed that take-out food is very popular for college students, with about two in three students consuming take-out food at least once a week and NL is associated with takeout food consumption.

In this study, we found that some factors were associated with take-out food consumption. Males ate take-out food more frequently than females. Similarly, a cross-sectional survey reported that males had higher fast-food consumption compared with females [31]. Although other study showed that medical students ate take-out food less frequently than non-medical students [17], we found that medical students consumed more take-out food than non-medical students. This may be associated with the fact that medical students are more likely to consume take-out food to save the time facing with heavy school workload. Our study showed that takeaway consumption was found to be higher among participants with higher monthly expenditures. Similar to the Australian study in which disadvantaged socioeconomic groups did not consume takeaway as much as advantaged groups [6, 32]. In Scotland, UK, consumption of takeaway food was significantly higher in the most deprived quintile [33]. This may be because the cost of take-out food varies from country to country, and the cost of eating take-out food in China may be higher than eating in the school cafeteria, and participants with higher monthly expenses have a higher economic level to consume take-out food. This study showed that participants who were less physically active consumed take-out food more frequently, physical activity promotes good health awareness among students, which may be a potential factor in reducing takeaway food consumption [34].

Our study used a comprehensive NL tool to examine a range of literacy cognition and skills. The results also demonstrated that poor NL had higher takeout food consumption. Moreover, the low nutrition skills domain was significantly associated with the high frequency of take-out food consumption, while the nutrition cognitive domain was not associated with takeout consumption. This may be due to the fact that take-out food consumption was associated with applying skills, interactive skills, and critical skills in the nutrition skills domain but no association with knowledge and understanding in the nutrition cognitive domain. Namdar et al. found that nutrition label reading skills and health decision-making ability were associated with fast food intake [35]. College students who regularly read nutrition labels were more likely to eat less fast food and added sugar [36]. Although prior studies showed that nutrition education increased nutrition knowledge, reduced the frequency of snacks and fried food [37], and had a positive effect on making healthier fast-food choices [38]. However, another study reported that nutrition education only increases nutrition knowledge, but failed to change eating behavior, which bring out the separation of knowledge and practice among college students [26]. These findings indicated that basic nutrition cognition could not directly change students’ health-related behavior such as take-out food consumption, but nutrition skills could do.

Several studies have shown the relationship between NL and dietary behavior, although adolescents had high dietary guidelines awareness or nutrition knowledge scale scores, they did not choose healthy foods or follow a Mediterranean diet [39, 40]. This suggests an urgent challenge for moving nutrition knowledge to the skills related to behavior changes, including making meal choices. College students are in a critical period of developing healthy eating behaviors [41]. Faced with misinformation about nutrition from the media, negative peer influence, and an unhealthy eating environment, college students’ perceptions of healthy behaviors are likely to be influenced by a lack of ability to identify scientific nutrition information and drive them to over-consume take-out foods [42]. Hence, college students must have adequate nutritional skills literacy, not only nutrition knowledge which is not enough to keep healthy diet.

NL is a crucial component for influencing food habits, Studies [43–45] reported that food literacy affected an individual’s ability to choose healthy food. Our study showed that participants with higher NL chose to eat more vegetable and fruit salads and less (Spicy) hot pots. Although eating take-out food once per week or more significantly lower mean fruit and vegetable consumption [3], unfortunately, take-out food is forming an increasingly important component of the diet among college students due to its selectivity and efficient delivery, even though it may not be as healthy as home-made and cafeteria food [6, 17]. But high NL might improve take-out food consumption and increase the probability of vegetable and fruit salads choice. This finding indicates that high NL is helpful to select health-friendly foods.

Our findings stressed that it was necessary to strengthen the multi-dimensional NL to achieve better nutrition education outcomes and further reduce takeout food consumption. There are some limitations in this study. First, it was not possible to establish the causal inference due to the cross-sectional study. Second, the study participants were from the city in the northern region of China only. Thus, our results could not represent the national college student population. Third, potential recall bias may be present due to the self-reported questionnaires, precise measurement on take-out food should be considered in future study.

## Conclusion

NL, especially in applying skills, interactive skills, and critical skills, is associated with consumption of take-out foods among college students. In addition, it also has an influence on types of take-out food consumption. Our findings emphasize that targeted interventions on nutritional skills literacy should be needed to improve dietary behaviors for student’s good health.

## Electronic supplementary material

Below is the link to the electronic supplementary material.

Supplementary Material 1

Supplementary Material 2

## Data Availability

The datasets analysed during the current study is available from the corresponding author on reasonable request.

## Abbreviations

NL: Nutrition literacy
