# Supplementary material for: High nutrition literacy linked with low frequency of take-out food consumption in chinese college students
Source: BMC Public Health. 2023 Jun 13;23:1132. doi: 10.1186/s12889-023-16078-9 (PMC10262486; doi:10.1186/s12889-023-16078-9)
Supplement: Supplementary file 1 — Supplementary Material 1 [file 12889_2023_16078_MOESM1_ESM.docx]

**Supplementary table 1** **Types of take-out food consumption**

| **Variables** | **N** | **%** |
| --- | --- | --- |
| ***Chinese dishes with rice*** |  |  |
| No | 998 | 46.9 |
| Yes | 1132 | 53.1 |
| ***Western fast food*** |  |  |
| No | 1647 | 77.3 |
| Yes | 483 | 22.7 |
| ***(Spicy) hot pot*** |  |  |
| No | 1424 | 66.9 |
| Yes | 706 | 33.1 |
| ***Barbecue skewers*** |  |  |
| No | 1800 | 84.5 |
| Yes | 330 | 15.5 |
| ***Pastries/ drinks*** |  |  |
| No | 1596 | 74.9 |
| Yes | 534 | 25.1 |
| ***Vegetable and fruit salad*** |  |  |
| No | 1920 | 90.1 |
| Yes | 210 | 9.9 |
