# Supplementary material for: High nutrition literacy linked with low frequency of take-out food consumption in chinese college students
Source: BMC Public Health. 2023 Jun 13;23:1132. doi: 10.1186/s12889-023-16078-9 (PMC10262486; doi:10.1186/s12889-023-16078-9)
Supplement: Supplementary file 2 — Supplementary Material 2 [file 12889_2023_16078_MOESM2_ESM.docx]

**Supplementary table 2 Items in the Nutrition Literacy Measurement Scale for Chinese Adults**

| **Knowledge** |
| --- |
| 1.1. Nutrition is an important material basis for health. |
| 1.2. Balanced diet and reasonable nutrition are important measures to prevent and control chronic diseases such as diabetes and hypertension. |
| 1.3. Drinking enough water every day is very important for health. |
| 1.4. Maintaining a balance between diet and physical activity is a reasonable way to lose weight. |
| 1.5. High-fat or processed meat products can increase the risk of chronic diseases. |
| 1.6. Steaming and boiling are healthier ways of cooking than frying and grilling. |
| 1.7. Refined white rice and flour are lacking in vitamins. |
| **Understanding** |
| 2.1. I have good reading and comprehension skills. |
| 2.2. I can easily understand the nutritional information delivered by new and traditional media. |
| 2.3. I have a good understanding of Chinese dietary guidelines and dietary pagodas. |
| 2.4. I have a good understanding of expert consensus regarding nutrition or dietary information. |
| 2.5. I can easily read the nutritional information on food packaging (such as energy, protein, sodium, and other nutrients). |
| **Obtaining** |
| 3.1. When encountering nutritional problems, I can get relevant advice from friends or family. |
| 3.2. I know where to find healthy diet information. |
| 3.3. I often read nutrition information transmitted through new media (eg, WeChat and microblogging) or watch nutrition-related programs. |
| 3.4. I can easily retrieve nutrition and diet information through the Internet. |
| 3.5. I have made friends with dietitians or nutritionists. |
| **Applying** |
| 4.1. I eat breakfast every day. |
| 4.2. I eat fruit every day. |
| 4.3. I often choose coarse cereals instead of refined rice or flour. |
| 4.4. I drink milk or dairy products every day. |
| 4.5. I rarely choose or prepare foods that are high in salt. |
| 4.6. I can easily judge the amount of common foods at home. |
| 4.7. I will buy or cook food appropriately to avoid waste. |
| 4.8. I will consciously choose diversified foods every day. |
| 4.9. I often buy foods based on nutrition information on food package. |
| 4.10. I will take nutrition into consideration when eating out. |
| 4.11. I don’t eat functional food rashly. |
| **Interactive** |
| 5.1. I often share nutritional information obtained from various channels with my family or friends. |
| 5.2. I have good communication skills. |
| 5.3. I often discuss a healthy diet with my family or friends. |
| 5.4. I can accept the reasonable nutrition and health ideas of my family or friends. |
| 5.5. If my family or friends are overweight and like high-fat foods, I will encourage them to change their eating habits. |
| 5.6. Although I am living in an environment where fresh food is not available within 15 minutes of walking distance, I can overcome this difficulty and insist on buying fresh food. |
| 5.7. I can easily reject unhealthy foods recommended by family or friends. |
| 5.8. Although I am surrounded by lots of unhealthy foods (high-salt, high-sugar, high-fat, fried, and barbecued foods), I can resist them and choose a healthy diet. |
| 5.9. I can make a reasonable diet plan for family or friends based on correct nutritional information. |
| **Critical** |
| 6.1. I can easily judge whether my daily diet is logical and reasonable. |
| 6.2. I can easily judge whether the nutritional information from new media is logical and reasonable. |
| 6.3. I can easily judge whether a diet is balanced with exercise. |
| 6.4. I can estimate the suitable food intake for maintaining healthy body weight. |
| 6.5. With a wide array of foods available, I can make appropriate choices according to their nutritional value and my health conditions. |
| 6.6. Despite sales promotion of functional food, I can make appropriate choices according to my health conditions. |

**Scoring Manual:** The nutrition cognitive domain included two dimensions: “knowledge” (7 items) and “understanding” (5 items). The nutrition skills domain consisted of four dimensions: “obtaining skills” (5 items), “applying skills” (11 items), “interactive skills” (9 items), and “critical skills” (6 items). Each item was scored ranging from 1 to 5 on a five-point Likert type scale (for knowledge dimension: 1=strongly disagreeable, 2= disagreeable, 3= neutral, 4=agreeable, 5= strongly agreeable; or for other dimensions: 1= strongly unmatched, 2=unmatched, 3= neutral, 4=matched, 5= strongly matched). Then the total scores were calculated from the 43 items, with a higher score indicating a higher NL level.
